# Melanoma cells with diverse invasive potential differentially induce the activation of normal human fibroblasts

**DOI:** 10.1186/s12964-022-00871-x

**Published:** 2022-05-10

**Authors:** Justyna Mazurkiewicz, Aleksandra Simiczyjew, Ewelina Dratkiewicz, Katarzyna Pietraszek-Gremplewicz, Michał Majkowski, Magdalena Kot, Marcin Ziętek, Rafał Matkowski, Dorota Nowak

**Affiliations:** 1 Department of Cell Pathology, Faculty of Biotechnology, University of Wroclaw, Joliot-Curie 14a, 50-383 Wrocław, Poland; 2 Faculty of Biotechnology, University of Wroclaw, Joliot-Curie 14a, 50-383 Wrocław, Poland; 3 Department of Oncology and Division of Surgical Oncology, Wroclaw Medical University, Plac Hirszfelda 12, 53-413 Wrocław, Poland; 4 Wroclaw Comprehensive Cancer Center, Plac Hirszfelda 12, 53-413 Wrocław, Poland

## Abstract

**Background:**

The tumor microenvironment consists of stromal cells, extracellular matrix, and physicochemical properties (e.g., oxygenation, acidification). An important element of the tumor niche are cancer-associated fibroblasts (CAFs). They may constitute up to 80% of the tumor mass and share some features with myofibroblasts involved in the process of wound healing. CAFs can facilitate cancer progression. However, their interaction with melanoma cells is still poorly understood.

**Methods:**

We obtained CAFs using conditioned media derived from primary and metastatic melanoma cells, and via co-culture with melanoma cells on Transwell inserts. Using 2D and 3D wound healing assays and Transwell invasion method we evaluated CAFs’ motile activities, while coverslips with FITC-labeled gelatin, gelatin zymography, and fluorescence-based activity assay were employed to determine the proteolytic activity of the examined cells. Western Blotting method was used for the identification of CAFs’ markers as well as estimation of the mediators of MMPs’ (matrix metalloproteinases) expression levels. Lastly, CAFs’ secretome was evaluated with cytokine and angiogenesis proteomic arrays, and lactate chemiluminescence-based assay.

**Results:**

Acquired FAP-α/IL6-positive CAFs exhibited elevated motility expressed as increased migration and invasion ratio, as well as higher proteolytic activity (area of digestion, MMP2, MMP14). Furthermore, fibroblasts activated by melanoma cells showed upregulation of the MMPs’ expression mediators’ levels (pERK, p-p38, CD44, RUNX), enhanced secretion of lactate, several cytokines (IL8, IL6, CXCL1, CCL2, ICAM1), and proteins related to angiogenesis (GM-CSF, DPPIV, VEGFA, PIGF).

**Conclusions:**

Observed changes in CAFs’ biology were mainly driven by highly aggressive melanoma cells (A375, WM9, Hs294T) compared to the less aggressive WM1341D cells and could promote melanoma invasion, as well as impact inflammation, angiogenesis, and acidification of the tumor niche. Interestingly, different approaches to CAFs acquisition seem to complement each other showing interactions between studied cells.

**Video Abstract**

**Supplementary Information:**

The online version contains supplementary material available at 10.1186/s12964-022-00871-x.

## Background

Melanoma is a skin cancer characterized by a high mortality rate [1]. Currently, several forms of targeted- and immunotherapies against melanoma are in clinical use, however, in the case of the majority of patients drug resistance develops rapidly. One of the factors that affect this phenomenon is the melanoma microenvironment, which consists of neighboring cells such as cancer-associated fibroblasts (CAFs), cancer-associated adipocytes, keratinocytes, and immune cells as well as physicochemical properties like acidification, oxygenation, and extracellular matrix (ECM) proteins [2, 3].

Fibroblasts are a major cellular component of the melanoma niche, which may constitute up to 80% of the tumor mass [4]. Normal fibroblasts can suppress melanoma development through the recruitment of immune cells in a paracrine way. Nevertheless, melanoma secretes different factors such as transforming growth factor-beta (TGFβ) and Nodal (a member of TGF superfamily), leading to activation of normal fibroblasts towards pro-tumorigenic CAFs, which are similar to myofibroblasts activated during the wound healing process [5–8]. Cancer-associated fibroblasts are characterized by increased expression of fibroblasts-activation protein-alpha (FAP-α), alpha-smooth muscle actin (α-SMA), and fibroblasts-specific protein 1 (FSP-1). However, activated fibroblasts are a heterogeneous group of cells and express these markers on different levels [9]. These cells may impact melanoma progression in various ways, e.g., by the production of matrix metalloproteinases, which facilitate melanoma invasion [10]. In addition, CAFs participate in the process of tumor angiogenesis through secretion of vascular endothelial growth factor and C-X-C-motif chemokine 12, which interacts with C-X-C-motif chemokine receptor 4 expressed on cancer cells and promotes endothelial cell recruitment [10, 11]. Activated fibroblasts also affect melanoma drug resistance development among others through the production of hepatocyte growth factor (HGF), basic fibroblasts growth factor (bFGF), and neuregulin 1, which support cell proliferation and tumor growth [10, 12, 13]. Moreover, the role of CAFs-secreted HGF in resistance acquisition is also described in the case of lung cancer [14].

In the area of the tumor microenvironment, cells may carry out anaerobic glycolysis, and as a result, an increased amount of lactate is produced, which is then transported to the extracellular space and contributes to raised external acidification [15]. The acidic niche leads to, important for cancer invasiveness, elevated adhesion [16], invasion [17], angiogenesis [18]. It corresponds also to the drug resistance [19, 20] and epithelial-mesenchymal transition (EMT) process through induction of extensive production of EMT-related proteins such as N-cadherin [21].

Recently, due to the essential role of CAFs in the formation of the tumor environment, this topic is widely studied, and some information concerning their function has been already discovered, but much—especially in the case of melanoma—remains unclear. Taking into account a great role played by CAFs in melanoma progression it is important to investigate interactions between these two cellular components of the tumor niche. Therefore, in our research we focused on the influence of melanoma cell lines derived from primary and metastatic tumors on normal human dermal fibroblasts (NHDF), considering their transformation into CAFs as well as cell properties associated with cancer progression.

## Methods

### Cell culture

Cancer-associated fibroblasts were differentiated from normal human dermal fibroblasts (Lonza) using four melanoma cell lines: A375 and Hs294T obtained from the American Type Culture Collection and WM1341D and WM9 cell lines purchased from Rockland Immunochemicals, Inc. Fibroblasts were cultured in FBM (Fibroblast Growth Basal Medium, Lonza) cell culture medium (supplemented with FGM™-2 SingleQuots™ from Lonza), whereas melanoma cells were grown in DMEM (Dulbecco’s Modified Eagle Medium) medium containing 4.5 g/l glucose and 1.5 g/l NaHCO_3_ supplemented with 10% fetal bovine serum (FBS), 2 mM glutamine, and antibiotics (10,000 U/ml penicillin, 10 mg/ml streptomycin, 25 µg/ml amphotericin B). Cells were cultured in 25 cm^2^ tissue culture flasks (VWR) at 37 °C in 5%CO_2_/95% humidified air and passaged twice a week using 0.25% trypsin/0.05% EDTA solution (IITD PAN, Wroclaw, Poland).

### Acquisition of the melanoma-conditioned medium

To obtain melanoma-conditioned media, used further for fibroblasts’ activation, melanoma cells were cultured in 75 cm^2^ tissue culture flasks. After they reached 70–80% confluence, cells were washed three times with PBS, and then fresh medium without FBS was added. Cells were cultured for 72 h, then the medium was collected, centrifuged at 1000×*g* for 15 min, and frozen at − 20 °C. Media obtained from at least three distinct biological repetitions were refrozen, pooled, filtered through 0.22 µm filters (Googlab), aliquoted, and stored in − 20 °C. We obtained combined media regularly and fibroblasts in subsequent biological replications were treated each time with a conditioned medium from another pooling.

### CAFs acquisition

**Fig. 1:**
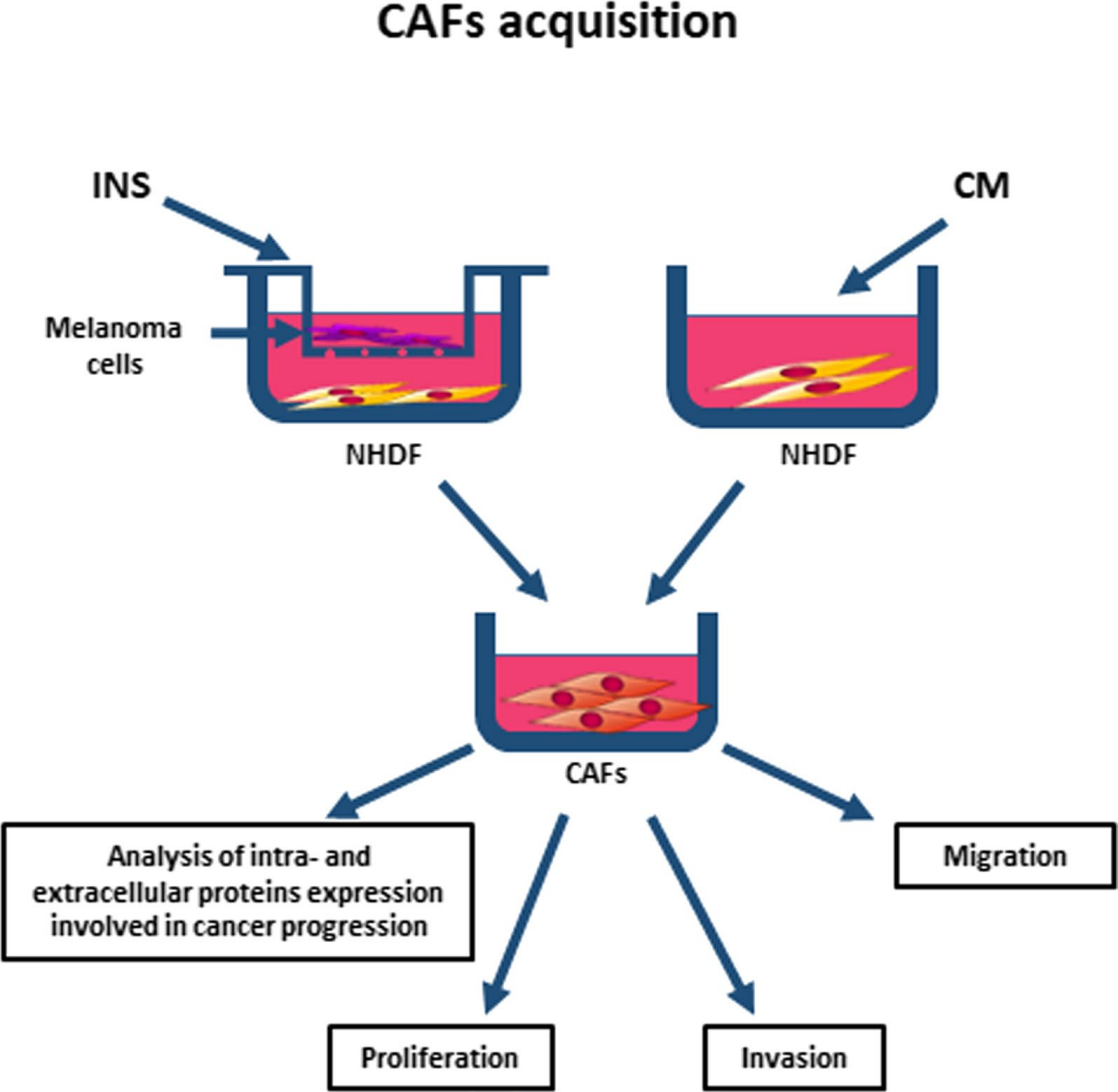
CAFs acquisition methods. Fibroblasts were cultured in the presence of melanoma cells cultured on Transwell inserts (INS) or with a melanoma-conditioned cell culture medium (CM) for 7 days. Next, cells were harvested and used for further experiments (analysis of migration, invasion, proliferation, level of intracellular proteins involved in cancer progression), or medium was changed for FBM without FBS, collected after 72 h and then used for the analysis of extracellular proteins level

CAFs were obtained from NHDF using transforming growth factor-beta (5 ng/ml, Corning) stimulation, or through culture in the presence of melanoma-conditioned media or indirect co-culture, with melanoma cells present on Transwell inserts (0.4 μm pores, Falcon) (Fig. 1). Fibroblasts were grown in differentiating conditions for seven days, with a single change of medium after four days of incubation. After this incubation time cells were collected and used for further experiments or—in the case of extracellular protein expression analysis—cells were washed three times with PBS and the culture media were changed to the fresh ones without FBS for another 72 h. Next, the media were harvested, centrifuged at 1000×*g* for 15 min, and frozen at − 20 °C.

Considering that in the experiments with melanoma-conditioned media, and in indirect co-culture, used media had to be suitable for two different types of cells, in both systems fibroblasts were grown in a combination of FBM and DMEM (1:1 ratio), therefore control for every experiment constitutes of fibroblasts cultured in such a medium. In the case of cells incubated with TGFβ, control cells were treated with TGFβ solvent (acetonitrile).

### Western blot analysis

Cells were grown in the presence of melanoma-conditioned medium (CM) or in co-culture with melanoma cells, then transferred on ice, washed three times with PBS, and lysates were harvested by addition of urea buffer (50 mM Tris, pH 7.4, 5% SDS, 8.6% sucrose, 74 mM urea, 1 mM dichlorodiphenyltrichloroethane) supplemented with protease and phosphatase inhibitors cocktails (Sigma). Media used for estimation of secreted protein levels were obtained as described in the CAFs acquisition section and concentrated using Amicon® Ultra-4 Centrifugal Filters (Merck Millipore). Protein concentration was determined using standard BCA (Bicinchoninic acid) procedure (Thermofisher). Samples of the identical amount of protein (10 μg—cell lysates, 5 μg—conditioned media) were separated by 10% polyacrylamide gel electrophoresis in the presence of sodium dodecyl sulfate (SDS-PAGE) as reported by Laemmli [22] and then transferred to nitrocellulose membranes, as reported by Towbin et al. [23]. Antibodies directed against α-SMA (Sigma, A5228), FAP-α (Sigma, 2191M1), pERK (Cell Signaling, 9101), ERK (Cell Signaling, 9102), IL6 (Abcam, ab6672), RUNX2 (Santa Cruz, 390351), CD44 (GeneTex, GTX628895), p38 (Cell Signaling, 8690), p-p38 (Cell Signaling, 4511), caveolin 1 (Cell Signalling, 3267) as well as goat anti-rabbit and anti-mouse antibodies conjugated with horseradish peroxidase (Cell Signaling Technologies) were utilized according to the manufacturer’s protocols. Immunoblots were developed using the Clarity Western ECL Substrate (Bio-Rad), then scanned with ChemiDoc (Bio-Rad), and analyzed with ImageLab Software (ver. 6.0, Bio-Rad). At least three independent experiments were conducted. Results were normalized to Ponceau S staining.

### 2D and 3D wound healing

Cells were seeded into ImageLock 96-well plates (IncuCyte ImgeLock, Sartorius) coated with Matrigel (1 mg/ml) and incubated for 24 h. Then, standardized wounds were generated in all wells simultaneously using Wound Maker™ (Essen Bioscience). To evaluate invasion, the cell-free zone and the cells were covered with an additional Matrigel (1 mg/ml) layer. Then, FBM medium was added on the cell layer directly (migration assay) or on the top of 3D Matrigel matrices (invasion assay). Phase-contrast time-lapse images were captured with a time interval of 2 h, utilizing a 10 × objective in an IncuCyte® Live-Cell Analysis System. Cells were allowed to cover the wound for 36 h. Representative results were further analyzed using IncuCyte® Scratch Wound Cell Migration Software Module (Sartorius). The relative wound density represents the increase in the area covered by the cells over time. Three repetitions of the experiments, each condition consisting of three replicates, were performed.

### Transwell invasion assay

Cell invasion tests were conducted using Transwell filters (8 µm pores, Falcon) placed in 24-well plates. Ahead of the experiment, CAFs obtained as previously described were starved for 24 h in serum-free FBM medium. Then, cells were seeded in medium without FBS onto each Transwell filter coated with Matrigel (1 mg/ml). In the bottom of the well medium containing 20% FBS served as a chemoattractant. After 24 h, non-invading cells and Matrigel present on the upper side of the filters were removed. Cells that invaded through the membrane were fixed with 4% formaldehyde; nuclei were stained with Hoechst 33342 and counted under the fluorescent microscope. The results of experiments are presented as a relative invasion factor (%). The number of control cells which invaded through the Transwell filters constitutes 100%. The experiments were performed three times, and each independent experiment was executed in triplicate.

### Proliferation assay

The Cell Proliferation Kit II (XTT) (Roche) was used according to the manufacturer’s protocol. Briefly, CAFs were seeded in 96-well plates. The XTT labeling mixture was added in parallel samples at time 0 (T0) and after 24 h (T24) of cell growth. After 3 h of incubation in 37 °C absorbances at 450 nm were measured and obtained values were background corrected. Based on the absorbance, the mean proliferation rate was calculated by dividing T24 by T0. Control cells’ proliferation was set as 100%. The experiments were conducted on at least three biological repetitions in triplicate for each condition.

### Fluorescent-gelatin degradation assay

The assays were performed according to the protocol described by Mazurkiewicz et al. [24]. Poly-L-lysine-coated coverslips (Corning) were washed with PBS and then fixed with 0.5% glutaraldehyde for 15 min. Next, the coverslips were placed on a 30 μl drop of gelatin labeled with fluorescein (FITC), incubated for 10 min, and rinsed with PBS. Then, sodium borohydride (5 mg/ml) was used for quenching the residual reactive groups. After washing with PBS, cells were seeded in 24-well plates containing a prepared before coverslips and incubated at 37 °C. After 8 h, cells were fixed with 4% formaldehyde, permeabilized with 0.1% Triton X-100, and stained with Alexa Fluor 568 phalloidin to visualize filamentous actin. Confocal images were captured using Leica SP8 confocal microscope and LAS X software (ver. 3.3.0, Leica). Places of degraded gelatin were visible as dark areas that lack fluorescence in the FITC channel in the bright green, fluorescent gelatin matrix.

Analysis was performed in Fiji software [25] employing scripts written in Fiji Macro language to automate the procedure. A single image subjected for analysis could contain a part of a single cell, a few parts of cells, a cell, a few cells, or a combination of the aforementioned situations. Image analysis was divided into two parts: in the first part the object of interest (i.e., at least a part of the cell) was segmented and its area was determined and assigned as “F-actin mask’s area” and the second part involved segmentation of the digested regions and their area determination. Gelatin-FITC proteolytic activity was defined as “digested area”/”F-actin mask’s area” × 100%. General workflow for the analysis of Gelatin-FITC digestion activity is presented in Additional file 3: Figure S3. Experiments were conducted in three repetitions.

### MMP14 activity assay

To assess MMP14 activity the SensoLyte 520 MMP14 Assay Kit (AnaSpec) was used. CAFs were obtained as previously described, then they were washed with PBS and harvested in assay buffer containing 0.1% Triton-X 100 and incubated for 10 min on ice. Further, samples were centrifuged at 2500×*g* for 10 min at 4 °C, and supernatants were transferred into fresh tubes. Protein concentration was measured using a BCA assay. Samples with the same amount of protein (ca. 10 µg) were incubated with an activator for 2 h at 37 °C. Next, the substrate was added, and the enzymatic reaction was performed for 30 min at 37 °C, and then stopped with a stop solution. The fluorescence of the product was measured (Ex/Em = 475/500 nm) using the GloMax Discover plate reader (Promega). For each experiment, the MMP14 activity was calculated compared to the control cells. Control was set as 100% of activity. Experiments were conducted in three repetitions; each condition was performed in two replicates.

### Gelatin zymography

The activity of secreted gelatinases was determined using cell-conditioned media. Following fibroblasts to CAFs transformation, cells were washed three times with PBS, and fresh medium without FBS was added, after 72 h of incubation in 37 °C, the medium was collected and concentrated using Amicon® Ultra-4 Centrifugal Filters (Merck Millipore). Next, protein concentration was determined using BCA (Thermofisher) assay, and cell-conditioned media were analyzed on SDS–polyacrylamide gels containing gelatin (1 mg/ml). The gels were stained with Coomassie Brilliant Blue R-250 (Sigma). Brighter stripes on gels were identified as the places of active gelatin degradation by MMPs. Gels were imaged with ChemiDoc (Bio-Rad) and densitometry of at least three biological replicates was performed using ImageJ software.

### Cytokine and angiogenesis arrays

To establish the composition of CAFs secretome a Proteome Profiler Human Cytokine and Angiogenesis Array Kits (R&D Systems) were utilized. The tests allow for the detection of 36 or 55 different cytokines and chemokines or angiogenesis-related proteins, respectively, thanks to antibodies spotted on the nitrocellulose membrane. The experiment was conducted according to the manufacturer’s protocol. Briefly, conditioned media collected from control cells, fibroblasts treated with CM from WM1341D, and WM9 (each sample containing 25 µg of protein) were mixed with biotinylated detection antibodies. These mixes were then added onto nitrocellulose membranes containing primary antibodies dots and incubated overnight. Next, the membranes were washed, and the signal was detected using streptavidin-HRP. A chemiluminescent signal was measured using ChemiDoc Imaging System (BioRad) and analyzed with ImageLab software (Bio-Rad). The densitometric signal was background corrected and then normalized to a reference spot (mean) for each membrane.

### Lactate secretion level

To evaluate the level of lactate secreted by CAFs, Lactate-Glo™ Assay (Promega) was used. In this test, lactate present in the medium undergoes oxidation catalyzed by lactate dehydrogenase, which is coupled with the reduction of NAD^+^. NADH, in turn, is used in pro-luciferin to luciferin reduction, utilized then to produce chemiluminescence. Analyzed media were collected as referred to in the CAFs acquisition section. Experiments were performed according to the manufacturer’s protocol. Luminescence was measured using the GloMax Discover plate reader (Promega). The luminescence value acquired from the control cells’ medium was set as 100%. Experiments were performed in three biological repetitions; each sample was conducted in triplicate.

### Statistical analysis

All data are given as means ± standard deviation (SD), and their significance was evaluated with GraphPad Prism 7 software applying one-way ANOVA followed by Tukey’s test or Kruskal–Wallis (in the case of gelatin-FITC digestion analysis).

## Results

In our experiments, we used four melanoma cell lines differing in invasiveness: derived from primary tumors—WM1341D and A375, as well as WM9 and Hs294T isolated from melanoma metastases. However, when we compared the influence of these cells on fibroblasts’ activity, we were taking into account not only their origin but also properties connected to cancer aggressiveness. Characterization of: WM1341D, A375, WM9, and Hs294T described earlier by Makowiecka et al. [26] revealed higher proteolytic activity, invadopodia formation ability, and a higher rate of migration and invasion in the case of A375, WM9, Hs294T cells in comparison to WM1341D cells. Therefore, in the further part of this manuscript, the WM1341D cell line is defined as the one with a less aggressive phenotype, and A375, WM9, Hs294T lines as highly aggressive cell lines.

### CAFs identification

First, three methods of fibroblasts differentiation were optimized, starting with the treatment of fibroblasts with TGFβ, which is known to activate this cell type [5]. However, there is a variety of melanoma-derived molecules, which may transform normal fibroblasts into CAFs, e.g., Nodal [6]. Therefore, we decided to use two other methods: fibroblasts (NHDF) cultured with melanoma-conditioned media (CM) or with melanoma cells present on the Transwell inserts (INS) (Fig. 1), and obtained CAFsCM and CAFsINS, respectively. To compare the effects exerted by primary and metastatic melanoma on normal fibroblasts, we performed experiments using four different melanoma cell lines—differing in the origin.

**Fig. 2:**
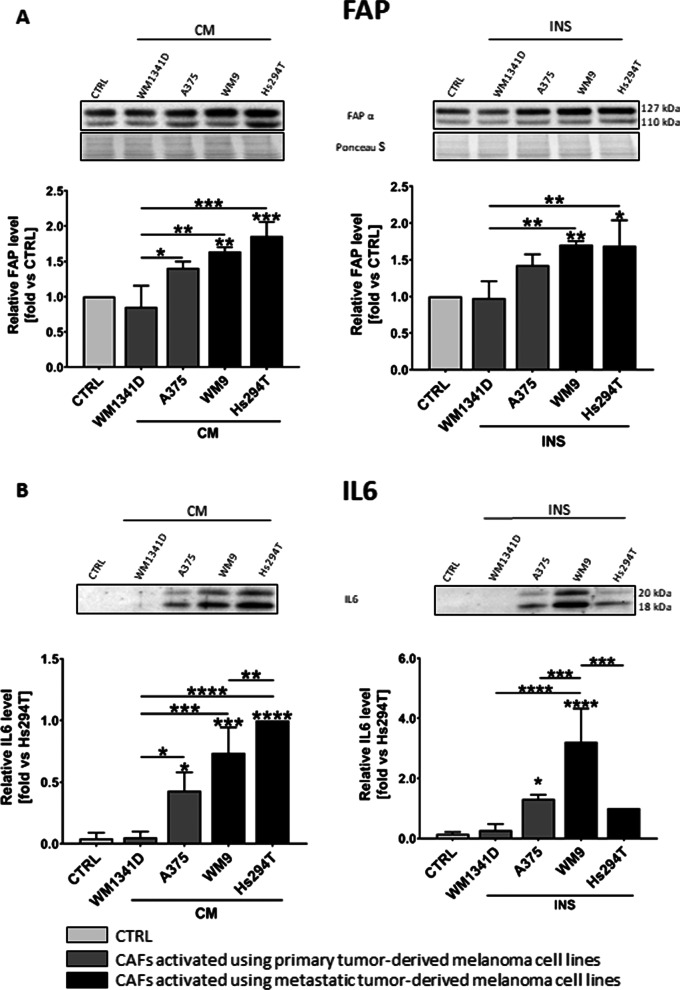
Identification of CAFs. Western Blot analysis of protein level of FAP (**A**) in cell lysates and IL6 (**B**) in media collected from activated fibroblasts cultured with melanoma-conditioned media (CM) or with melanoma cells growing on Transwell inserts (INS). Signal was normalized to total protein content assessed by Ponceau S staining. Control (CTRL) constitutes fibroblasts cultured in FBM:DMEM (1:1 ratio) media analogous to tested cells. In the case of IL6, due to the lack of signal in CTRL, the fold is set vs signal from the last lane (Hs294T). The mean of at least three biological repetitions ± SD is shown. Asterisks indicate statistically important differences between control cells and CAFs or between different types of CAFs. The significance level was set at *p* ≤ 0.05 (*), *p* ≤ 0.01 (**), *p* ≤ 0.001 (***), and *p* ≤ 0.0001 (****)

We have assessed activation of fibroblasts based on the presence of markers typical for CAFs (α-SMA, FAP-α) using Western Blot analysis. Fibroblasts activated by TGFβ were characterized by a high α-SMA level (Additional file 1: Fig. S1), whereas fibroblasts cultured with melanoma CM and from indirect co-culture displayed a high FAP-α level (Fig. 2A). Cells treated with TGFβ did not exhibit changes in activation of proteins connected to cancer progression and involved in the mitogen-activated protein kinase (MAPK) pathway, which abnormal regulation may lead to increased or uncontrolled cell proliferation and resistance to apoptosis of cancer cells. Research into the MAPK pathway has shown it to be important for some cancers, expressed as an increase in phosphorylation of extracellular signal-regulated kinase (ERK) (Additional file 1: Fig. S1). This effect was observed in the case of CAFs co-cultured with melanoma and melanoma CM-treated CAFs (Additional file 1: Fig. S1). Since CAFs co-cultured with melanoma (INS) and cultured in the presence of melanoma conditioned media (CM) seem to better reflect in vivo conditions, thus further experiments were performed using only the model of CAFsCM and CAFsINS.

We also confirmed the transformation of fibroblasts towards CAFs through the observation of elevated extracellular interleukin 6 (IL6) level (Fig. 2B), which is characteristic of this type of cells. Moreover, fibroblasts cultured in the presence of metastatic melanoma secretome exhibited a higher level of FAP-α (in both experimental models—CAFsINS, CAFsCM), and IL6 (only in the case of CAFsCM) in comparison to cells incubated with melanoma cells derived from primary tumors (Fig. 2).

### CAFs migration and invasion

**Fig. 3:**
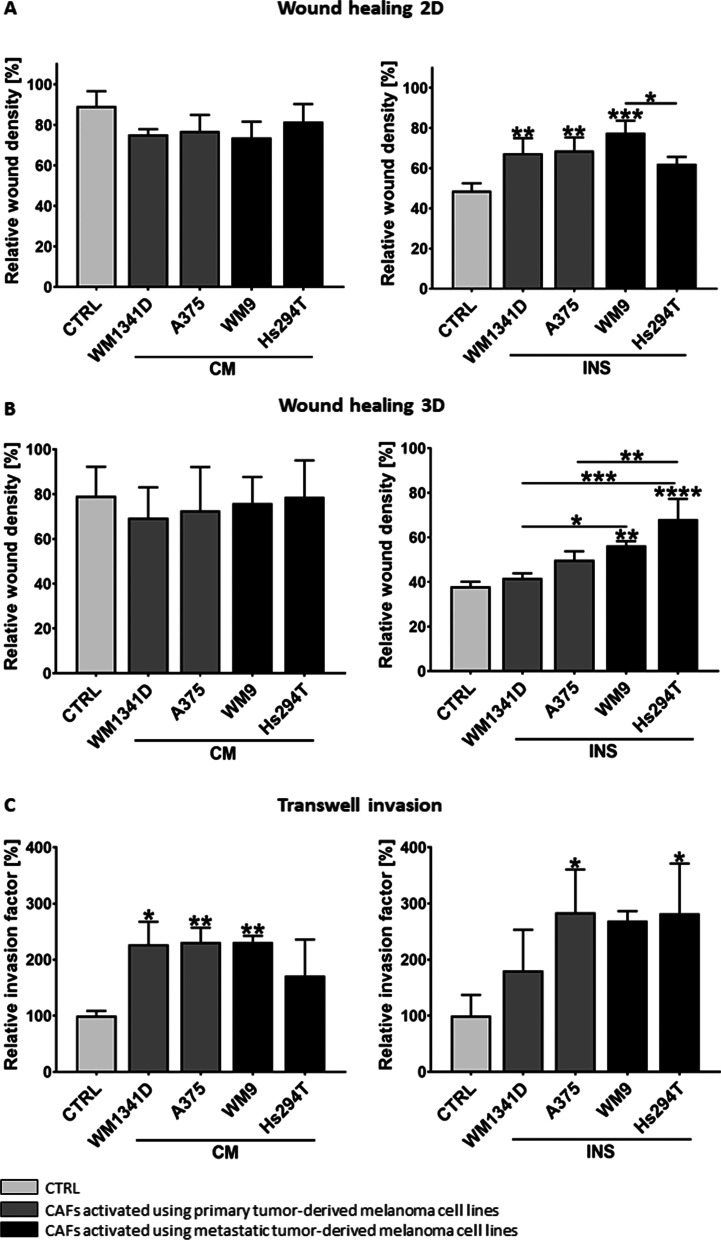
Analysis of CAFs’ migratory and invasive abilities obtained in culture with melanoma-conditioned media (CM) or melanoma cells on Transwell inserts (INS). Control (CTRL) constitutes of fibroblasts cultured in FBM:DMEM (1:1 ratio) analogous to tested cells. To investigate migration and invasion **A** 2D and **B** 3D wound healing assays were performed. Relative wound density—determined using IncuCyte® Live-Cell Analysis System—is shown. Transwell invasion tests were also conducted (**C**). The number of control cells that invaded was set as 100%. The relative invasion factor was calculated. All experiments were conducted three times, each performed in triplicate and the mean ± SD is shown. Asterisks indicate statistically important differences between control cells and CAFs or between different types of CAFs. The significance level was set at *p* ≤ 0.05 (*), *p* ≤ 0.01 (**), *p* ≤ 0.001 (***), and *p* ≤ 0.0001 (****)

In the next step, CAFs were characterized concerning the processes involved in metastasis formation. Migration-imitating cell movement in two-dimensional (2D) conditions, e.g., on the basement membrane surface was estimated using directional migration scratch assay. We observed a higher migration rate in the case of CAFsINS in comparison to normal fibroblasts (Fig. 3A), whereas we did not notice any difference between examined CAFsCM and control conditions. To evaluate the invasive ability of fibroblasts, we performed a 3D wound healing assay, in which cells were placed between two layers of Matrigel imitating basement membrane and, thus, in vivo conditions. The relative wound density was higher in CAFsINS co-cultured with metastasis-derived cell lines (WM9, Hs294T) in comparison to control cells and fibroblasts cultured in the presence of primary melanoma cell lines (WM1341D and A375) (Fig. 3B). We did not observe any significant differences in the case of CAFsCM invasion.

Further, invasion assays through Transwell inserts were conducted, where cells invaded through the Matrigel towards a chemoattractant added to the medium. A significant increase in the invasion capacity was observed in the case of CAFsCM and CAFsINS in comparison to control fibroblasts (Fig. 3C). To confirm that the observed changes in CAFs migration and invasion abilities are not the result of their enhanced cell division rate, proliferation assays were performed indicating no differences between CAFs and control cells (Additional file 2: Fig. S2).

### Proteolytic activity of CAFs

**Fig. 4:**
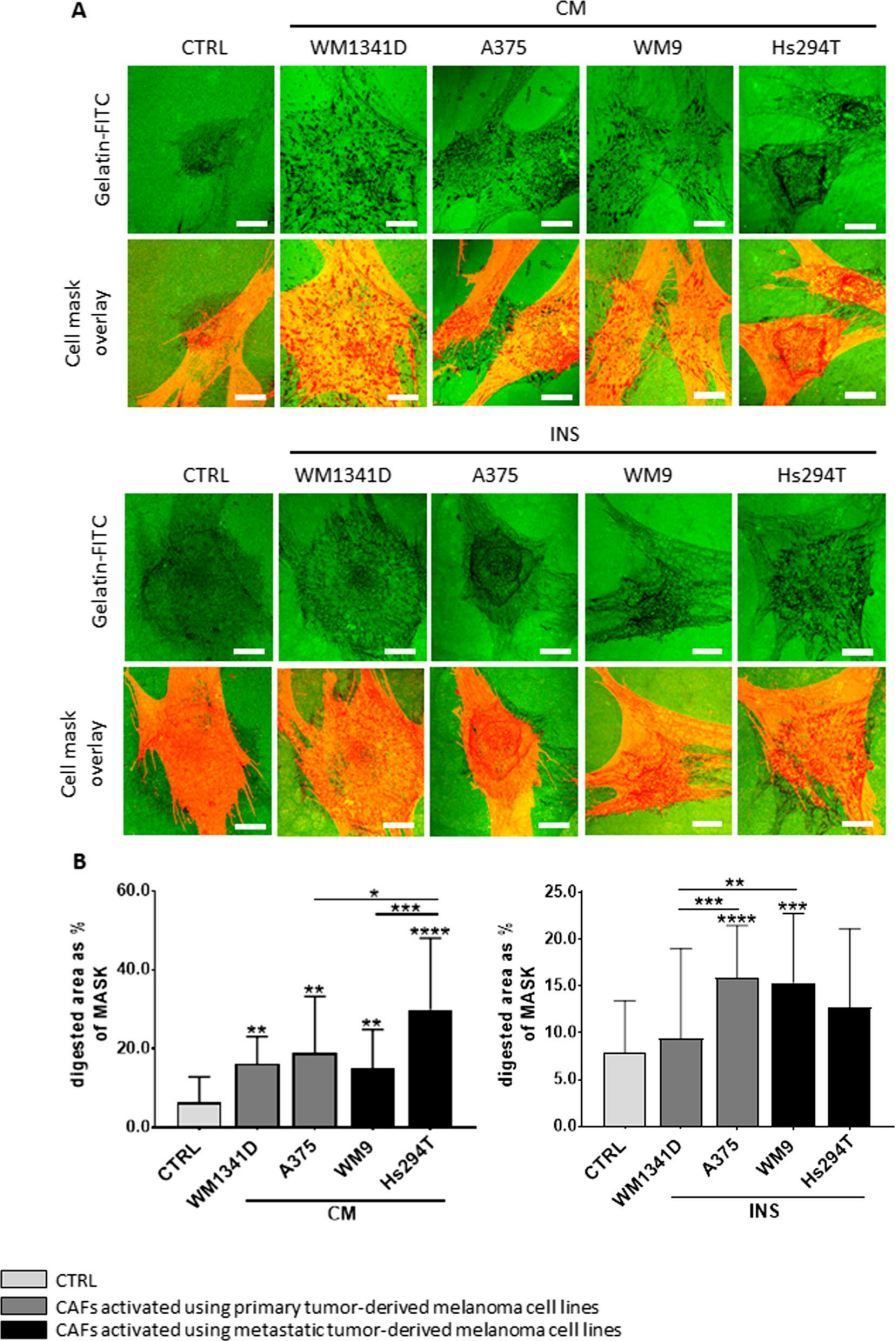
The influence of melanoma cells on the proteolytic activity of fibroblasts. CAFs obtained using melanoma-conditioned media (CM) and melanoma cells on Transwell inserts (INS) were seeded on coverslips coated with gelatin-FITC (green). Control (CTRL) constitutes of fibroblasts cultured in FBM:DMEM (1:1 ratio) analogous to tested cells. **A** After 8 h of incubation, cells were fixed and stained using phalloidin to visualize F-actin, which then were used to create a cell mask. Areas of gelatin degradation are visible as dark holes on a green background. Scale bar—25 µm. **B** Digestion areas were determined in Fiji software. F-actin staining was used to create a cell mask and determine its area. Asterisks indicate statistically important differences between control cells and CAFs or between different types of CAFs. The significance level was set at *p* ≤ 0.05 (*), *p* ≤ 0.01 (**), *p* ≤ 0.001 (***), and *p* ≤ 0.0001 (****)

Proteolytic enzymes secreted by cells during invasion digest ECM proteins and, in this way, form a pathway used by cancer cells to move through the tissue. Due to the fact, that we observed differences in invasion rate between normal fibroblasts and CAFs, we also compared their proteolytic activity. To realize this purpose a gelatin-FITC degradation assay was conducted, in which cells digested fluorescently-labeled gelatin leading to the appearance of black spots on a fluorescent green background. In addition, we stained filamentous actin (F-actin) using phalloidin conjugated to Alexa Fluor 568 to visualize actin cytoskeleton organization, including the occurrence of podosomes—cellular protrusions involved in cell movement. We detected changes in proteolysis patterns between normal fibroblasts and CAFs (Fig. 4A). Moreover, it was observed that CAFs digested gelatin-FITC to a higher extent than control fibroblasts (Fig. 4B).

**Fig. 5:**
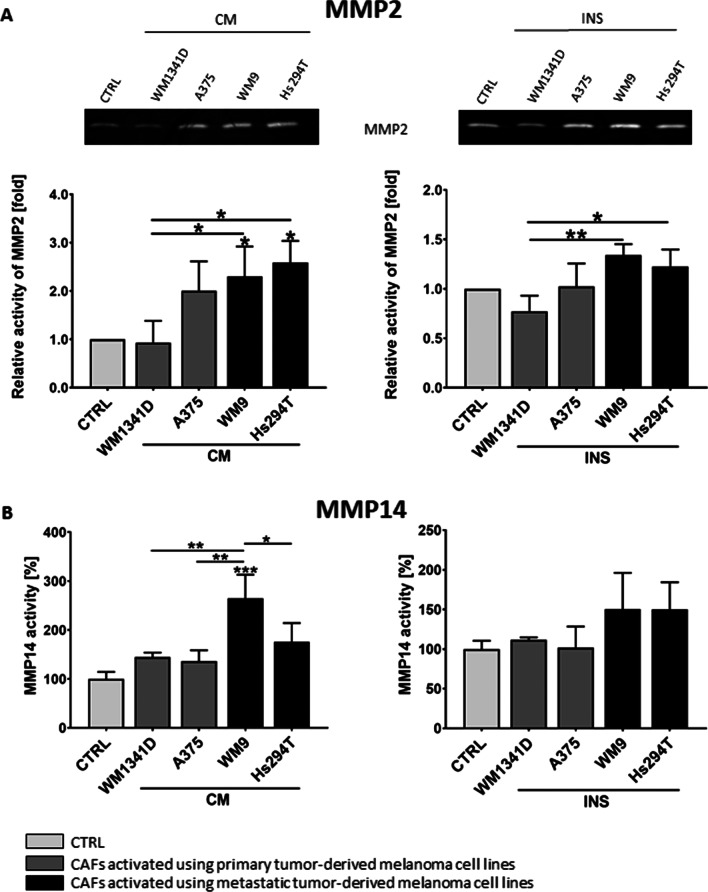
Matrix metalloproteases’ activity in CAFs. **A** Images of representative gelatin zymography gels performed on media collected from CAFs. The mean of densitometry quantification of at least three independent repetitions of gelatin zymography ± SD is shown. **B** As MMP14 is a membrane metalloprotease, its activity was measured in cell lysates collected from CAFs, using a fluorimetric activity assay. The data represent the mean MMP14 activity of three independent measurements ± SD. In both experiments, CAFs were obtained from fibroblasts cultured with melanoma-conditioned media (CM) or with melanoma cells on Transwell inserts (INS). Control (CTRL) constitutes of samples collected from fibroblasts cultured in a composition of FBM:DMEM (1:1 rate) medium. Asterisks indicate differences between control cells and CAFs or between different types of CAFs. The significance level was set at *p* ≤ 0.05 (*), *p* ≤ 0.01 (**) and *p* ≤ 0.001 (***)

The changes observed in gelatin-FITC degradation in CAFs compared to normal fibroblasts prompted us to further analyze the composition of proteases secreted by examined cells. We conducted gelatin zymography using conditioned cell culture media from fibroblasts and CAFs, in which digestion of the gelatin by proteases is visible as a transparent band on a dark background. We detected a higher MMP2 activity in CAFs media than in media collected from fibroblasts culture. Moreover, we have noted that metastatic melanoma led to a higher activity of MMP2 in CAFs than fibroblasts cultured in the presence of melanoma derived from primary tumors (Fig. 5A). Next, we performed an MMP14 activity assay and observed its higher activity in the case of fibroblasts derived from a culture with metastatic melanoma media in comparison to control cells (Fig. 5B). In the case of CAFsINS, we also observed such a tendency, but the results were not statistically significant.

**Fig. 6:**
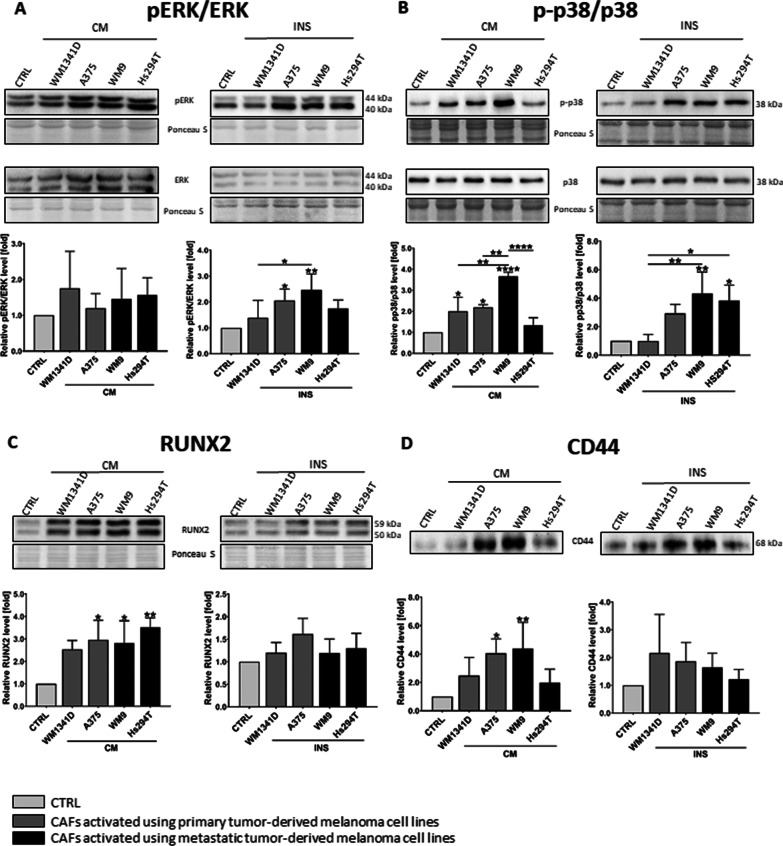
The expression level of proteins connected to MMPs expression. CAFs were obtained from fibroblasts cultured with melanoma-conditioned media (CM) or with melanoma cells on Transwell inserts (INS). Control (CTRL) constitutes of fibroblasts cultured in a composition of FBM:DMEM (1:1 rate) medium. Representative membranes from Western Blot analysis of regulators of MMPs expression performed using CAFs lysates (pERK, ERK (**A**), p38 (**B**), RUNX2 (**C**)) and CAFs’ conditioned media (CD44 (**D**)). Signal was normalized to total protein content assessed by Ponceau S staining. At least three repetitions were performed. Asterisks indicate statistically important differences between control cells and CAFs or between different types of CAFs. The significance level was set at *p* ≤ 0.05 (*), *p* ≤ 0.01 (**), and *p* ≤ 0.0001 (****)

To further unravel the basis of the increased MMPs’ activity, we analyzed proteins related to cell motility and signaling such as the MAPK pathway [27]. Western Blot analysis of CAFs’ lysates revealed a higher phosphorylation rate of ERK and p38 (Fig. 6A, B) in CAFs, compared to normal fibroblasts.

CD44 (Cluster of Differentiation 44) is a transmembrane hyaluronic acid receptor that can act as the organizing platform for MMPs and can be cleaved by proteases resulting in extra- and intracellular forms, where the latter one was reported to bind to runt-related transcription factor 2 (RUNX2) and work as a co-transcriptional factor for metastatic-related genes, e.g., MMP9 in prostate cancer [28, 29]. Therefore, we decided to estimate the level of CD44 in conditioned media and RUNX2 in lysates obtained from CAFs. We conducted Western Blot analysis and demonstrated a higher level of RUNX2 and CD44 in CAFs compared to fibroblasts (Fig. 6C, D), although these differences are statistically significant only in the case of CAFsCM.

### Characterization of CAFs’ secretome

**Fig. 7:**
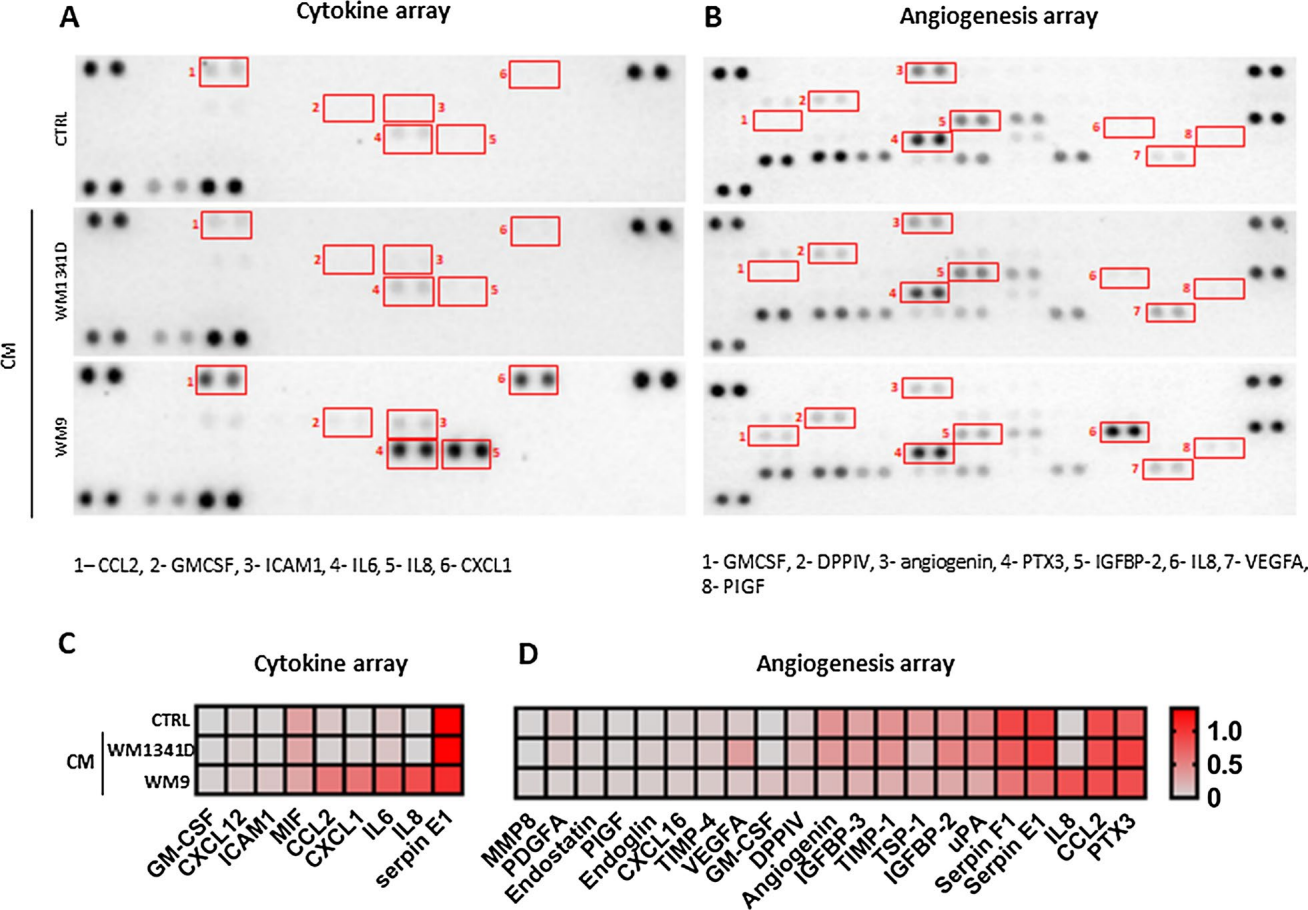
CAFs’ secretome analysis. To identify proteins secreted by CAFsCM’, conditioned media were used for cytokine (**A**, **C**) and angiogenesis (**B**, **D**) arrays. Based on obtained signals (**A**, **B**) quantitative analysis (**C**, **D**) was performed. Densitometric data were normalized to reference spots and are presented in a form of heatmaps, where darker red indicates a higher increase in signal. Abbreviations: CCL2, C-C Motif Chemokine Ligand 2; GM-CSF, Granulocyte–macrophage colony-stimulating factor; ICAM1, Intercellular Adhesion Molecule 1; IL6, interleukin 6; CXCL1, C-X-C Motif Chemokine Ligand 1; DPPIV, Dipeptidyl‐peptidase VI; PTX3, Pentraxin 3; IGFBP-2, Insulin-Like Growth Factor Binding Protein 2; VEGFA, vascular endothelial growth factor A; PIGF, Placental growth factor A; MIF, macrophage migration inhibitory factor; PDGF A, Platelet-derived growth factor; TIMP-4, tissue inhibitor of metalloproteinases 4; TSP-1, Thrombospondin 1; uPA, urokinase-type plasminogen activator

In our research, we focused on features of CAFs connected to cancer progression, which includes not only proteolysis but also angiogenesis and inflammation processes. Taking into account, that cells present in the tumor niche communicate with each other through direct interactions and in a paracrine manner [30], we selected representative melanoma cell lines derived from primary (WM1341D) and metastatic (WM9) tumors for fibroblasts activation, and performed profiling of human cytokine and angiogenesis factors in CAFs’ media. In Fig. 7 secreted proteins whose expression differs between control and CAFs are shown. In the case of cytokines, the secretion of at least five of them (i.e., CCL2 (i.e., C-C Motif Chemokine Ligand 2), CXCL1 (C-X-C Motif Chemokine Ligand 1), IL6, IL8, ICAM1 (Intercellular Adhesion Molecule 1)) is specifically induced in CAFs obtained using metastatic melanoma cells (WM9) and undetected in primary melanoma-derived CAFs (Fig. 7A, C). On the other hand, the treatment with WM9-conditioned medium seems to reduce angiogenic mediators secretion by CAFs with an exception of IL8, Granulocyte–macrophage colony-stimulating factor (GM-CSF), placental growth factor (PIGF), dipeptidyl‐peptidases IV (DPPIV), while it is maintained or even elevated (e.g., vascular endothelial growth factor A (VEGFA), Insulin-Like Growth Factor Binding Protein 2 (IGFBP-2) and Pentraxin 3 (PTX3)) in the case of WM1341D-derived CAFs.

**Fig. 8:**
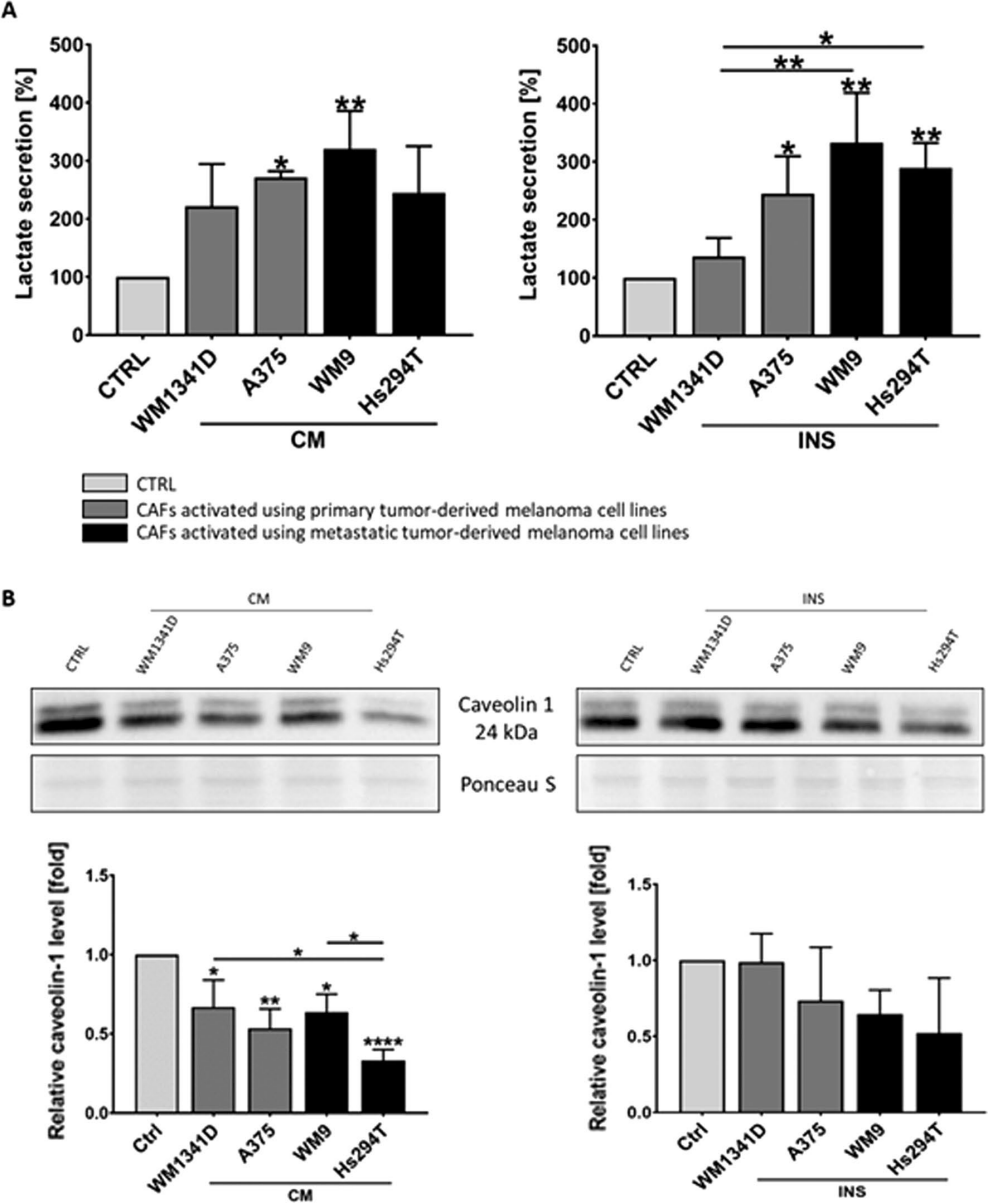
Influence of melanoma cells on lactate secretion (**A**) and caveolin 1 level (**B**) in CAFs. CAFs were obtained from fibroblasts cultured with melanoma conditioned media (CM) or with melanoma cells on Transwell inserts (INS) for 7 days. Control (CTRL) constitutes of medium collected from fibroblasts cultured in a composition of FBM:DMEM (1:1 rate) medium. The level of secreted lactate was measured using a chemiluminescent reaction (**A**), whereas caveolin 1 level was estimated in western blotting analysis (**B**). The mean of at least three independent repetitions ± SD is shown. Asterisks indicate statistically important differences between control cells and CAFs or between different types of CAFs. The significance level was set at *p* ≤ 0.05 (*), *p* ≤ 0.01 (**), and *p* ≤ 0.0001 (****)

An important factor, which modifies the cancer microenvironment and has an influence on cancer progression is also extracellular acidification induced by lactate secretion. Therefore, we estimated the level of lactate secreted by fibroblasts, under the influence of melanoma cells. Analysis of lactate level in CAFs’ media revealed extensive secretion of this metabolite after activation of fibroblasts by melanoma cells. Furthermore, in the case of CAFsINS, we observed higher lactate levels in CAFs co-cultured with more aggressive melanoma (A375, WM9, Hs294T) cells than those, which were grown in the presence of less aggressive WM1341D cells (Fig. 8A). Furthermore, metabolic reprogramming of CAFs, which results in increased lactate secretion can be triggered by downregulation of caveolin 1 (Cav-1) expression [31, 32]. We performed Western blot analysis and established that the level of caveolin-1 was reduced in CAFs compared to control cells (Fig. 8B).

## Discussion

Fibroblasts are the major component of the tumor niche, especially in the case of skin cancer. At the tumor onset, they may suppress cancer development by induction of inflammation processes [34]. However, cancer cells are able to transform adjacent fibroblasts into cancer-associated ones, which are more supportive for their survival [13]. CAFs secrete several factors involved in cancer progression such as MMPs, cytokines, and growth factors [2, 10, 35]. Taking into account the great role played by CAFs in the modulation of the tumor microenvironment and cancer progression, it is important to investigate the molecular basis of the interactions between these two types of cells. Therefore, in this research, we focused on the influence exerted by melanoma cells on fibroblasts’ functions.

We established two different methods of CAFs acquisition based on commonly utilized systems: using melanoma-conditioned medium [36] and with melanoma cells present on Transwell inserts [37, 38]. Both approaches worked well, leading to fibroblasts’ activation identified by high FAP-α and IL6 levels, which are markers of this type of cells described in the literature [34, 39, 40]. In the first model, fibroblasts were treated with melanoma-conditioned media. This method might be characterized by a greater reproducibility of the obtained results between biological replications of experiments because only fibroblasts are a variable factor here (they may slightly change their phenotype from passage to passage). Despite the conditioned media are each time combined from several biological repetitions, their composition is not so fluctuating. In the case of the indirect co-culture, the reproducibility of the results may be lower, because both the fibroblasts and the melanoma cells can undergo some phenotypic changes with subsequent passages. Due to that, we observed lower standard deviation and, thus, statistically significant differences in the level of MMP2, MMP14, RUNX2, or CD44 in CAFs obtained by incubation with conditioned media, but not by co-culturing with melanoma cells present on Transwell inserts. However, a major advantage of the CAFsINS culture method is that cells grow in one environment and influence each other, modulating their responses, what may reflect conditions more similar to those prevailing in vivo. This may be the reason why we observed higher migratory and invasive abilities in CAFs obtained by indirect co-culture with melanoma cells, than those treated with melanoma CM. Due to the aforementioned distinctions, we decided that it was more appropriate to use both of these models in our research. 

To investigate the differences in the effects exerted by primary tumor- and metastasis-derived melanoma cells on normal fibroblasts activity, we utilized four different melanoma cell lines: WM1341D, A375 (primary tumor-derived), and WM9, Hs294T (metastasis-derived). However, these cell lines differ not only due to their origin but also in aggressiveness. Therefore, during the analysis of our results, we focused on both aspects. We established that metastasis-derived melanoma stimulates motility and proteolytic activity of CAFs to a greater extent than primary tumor-derived melanoma cell lines. However, a higher level of activation and lactate secretion was triggered in CAFs by highly aggressive A375, WM9, Hs294T cell lines (where A375 is derived from the primary tumor) than by less aggressive WM1341D cells (also derived from the primary tumor). In this way, we have demonstrated that not only the origin but also aggressiveness of melanoma cells is important for CAFs activation.

Next, we analyzed the impact of melanoma on fibroblasts in terms of cancer progression. We detected changes in fibroblasts’ migration, invasion, and proteolytic activity—features significant for metastasis formation. Increased proteolysis of ECM in the tumor microenvironment may facilitate cancer cells’ invasion, as in this way, they form a pathway that can be used by other cells to move through the surrounding tissue [41–43]. A majority of studies concerning crosstalk between CAFs and melanoma have focused only on changes in melanoma motility under the influence of CAFs [38, 44–46]. Because cancer cells may move collectively together with fibroblasts [47–50], it is also important to investigate the migratory abilities of CAFs. Our results, indicating increased migration of CAFs compared to normal fibroblasts, are consistent with the study of the motility of CAFs treated with melanoma-derived exosomes [51, 52] and fibroblasts isolated from gastric cancer [53]. Furthermore, we demonstrated activation of the MAPK pathway (upregulated phosphorylation of ERK and p38) in CAFs, which may lead to increased proliferation and migration of these cells and, thus, enhance melanoma progression [54]. We also observed an enhanced level of RUNX2 and CD44—proteins connected to MMPs’ expression. In prostate cancer, an intracellular form of CD44—released upon extracellular domain cleavage—is transported into the nucleus, where it can bind to RUNX2 leading to its activation and subsequent increased MMPs expression [29]. It has not been yet reported in the case of melanoma-associated fibroblasts. We detected upregulation of all above-mentioned proteins from this pathway (CD44, RUNX2, MMPs) in CAFs, nevertheless, a conclusion that in CAFs this pathway is also present would require further experiments.

As secretome is an important part of the crosstalk between cells, we decided to analyze secretion patterns of CAFs by focusing on the molecules involved in the regulation of inflammation and angiogenesis using Cytokine and Angiogenesis Arrays. We detected higher levels of various cytokines, i.e., CCL2, IL8, CXCL1, GM-CSF, ICAM1, IL6; factors influencing angiogenesis, namely DPPIV and VEGF, and reduced secretion of angiogenin in CAFs compared to control cells. The increase in CCL2, IL8, and CXCL1 was already observed in melanoma-associated fibroblasts. Moreover, it was shown that highly invasive melanoma cells affect the expression of these proteins to a greater extent than less invasive melanoma [55]. Secretion of GM-CSF, DPPIV, ICAM1 and PIGF by melanoma-associated fibroblasts was not reported before.

It should be mentioned that GM-CSF is involved in immune cell activation, and it was tested as a form of monotherapy against melanoma. However, the drug based on GM-CSF was rejected at the stage of clinical trials due to low treatment efficiency [56]. On the other hand, in a melanoma mouse model, a positive correlation between GM-CSF expression and tumor growth has been observed [57]. As we detected this protein in CAFs’ medium, it is possible, that CAF-derived GM-CSF could influence melanoma progression.

Another protein, detected in CAFs secretome was ICAM1, which is present on the surface of many hematopoietic and nonhematopoietic cells and may act as an adhesive ligand for the integrins, indicating its involvement in cell-substrate adhesion [58]. Interestingly, pERK is described to activate the expression of ICAM1 in rat fibroblasts [59]. Moreover, it was shown that in melanoma patients, the presence of ICAM1 correlates with a worse prognosis [60]. In turn, PIGF is a member of the VEGF family. According to our best knowledge, secretion of PIGF by melanoma-associated fibroblasts has not been reported before. However, Aoki et al. demonstrated, that CAFs stimulate invasion of intrahepatic cholangiocarcinoma by PIGF secretion [61]. On the other hand, we observed a lower level of angiogenin in CAFs compared to control cells, which was shown by Song et al. to be crucial for bFGF-induced melanoma cell proliferation [62].

We also detected higher lactate levels in CAFs compared to control cells. Secreted lactate may cause tumor niche acidification, which in turn activates invasion and angiogenesis in cancer cells and thus corresponds to cancer progression [21]. Increased lactate secretion may stem from reduced caveolin 1 level in CAFs. Interestingly, data obtained by Wu et al. showed that loss of stromal Cav-1 expression was also linked to poor survival and increased metastatic potential in melanoma [33]. It seems that melanoma cells induce fibroblasts to enhance extracellular acidification that in turn might support tumor progression.

Taking into account that CAFs have a great impact on cancer progression, additional CAFs-directed therapy for melanoma suffering patients would be worth considering [40, 63]. It is especially important in the case of patients with metastases, as our research shows that fibroblasts are activated to a greater extent by metastatic melanomas cells compared to the ones derived from the primary tumor.

## Conclusions

**Fig. 9:**
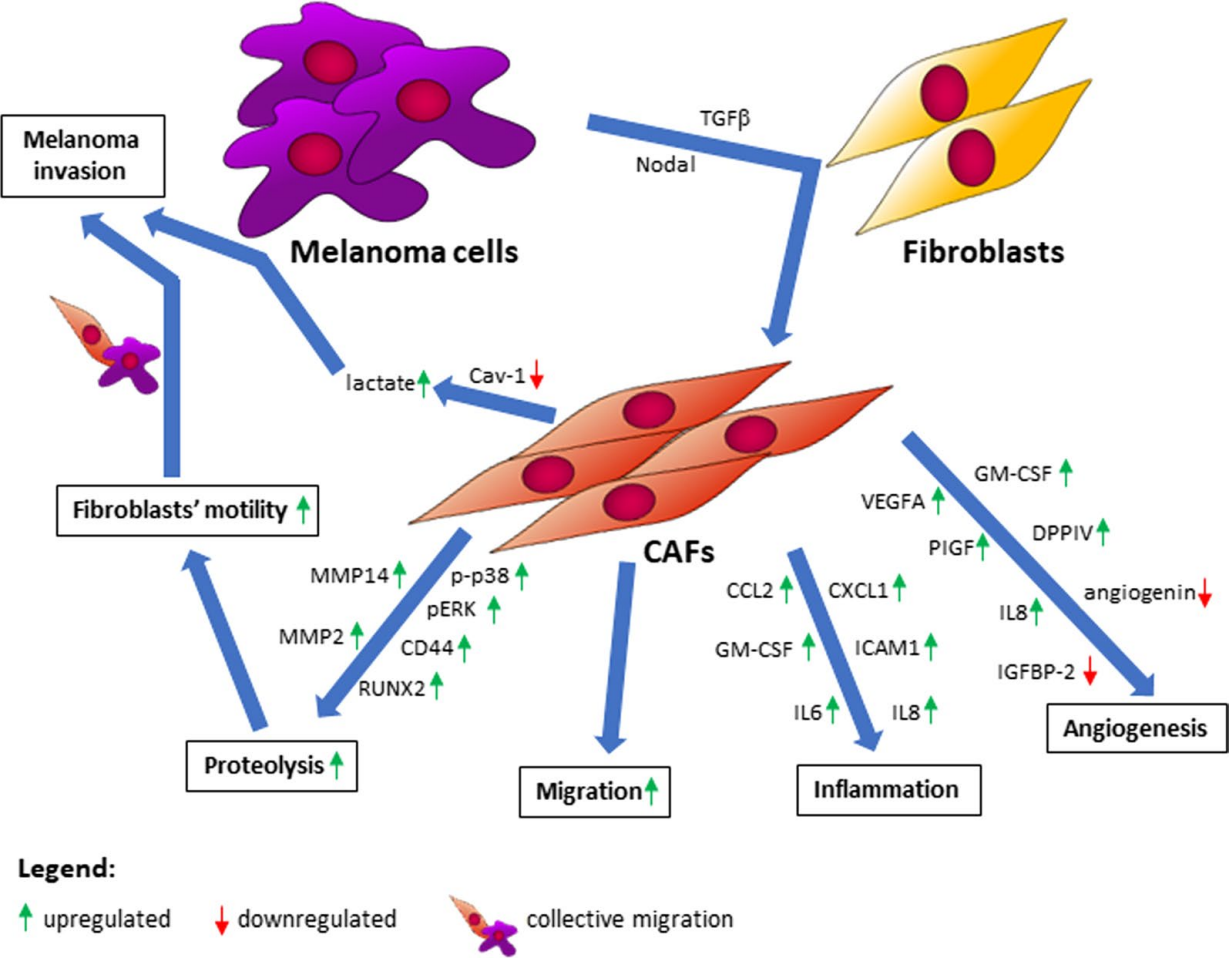
Crosstalk between melanoma and CAFs. Abbreviations: MMP, matrix metalloproteinase; p-p38, phosphorylated-p38; pERK, phosphorylated extracellular signal-regulated kinase; CD44, cluster of differentiation 44; RUNX2, runt-related transcription factor 2; CCL2, C-C Motif Chemokine Ligand 2; CXCL1, C-X-C Motif Chemokine Ligand 1; GM-CSF, Granulocyte–macrophage colony-stimulating factor; ICAM1, Intercellular Adhesion Molecule 1; IL6, interleukin 6; VEGFA, vascular endothelial growth factor A; PIGF, Placental growth factor; IGFBP-2, Insulin-Like Growth Factor Binding Protein 2; DPPIV, Dipeptidyl‐peptidase VI; TGFβ, transforming growth factor-beta; Cav-1, caveolin 1; CAFs, cancer-associated fibroblasts

Concluding, the use of two different models imitating the relationship between melanoma-associated fibroblasts and melanoma cells within the microenvironment seems to be appropriate to gain a broader view of these cells’ crosstalk. Furthermore, the influence of melanoma cells on fibroblasts differs depending on cancer cells’ origin and their aggressiveness. In selected experiments, primary tumor-derived melanoma cells affected CAFs to a lesser extent compared to metastatic. Moreover, in some cases less aggressive WM1341D cells led to weaker responses of fibroblasts than highly aggressive melanoma cell lines (A375, WM9, Hs294T). We observed higher proteolytic activity and level of proteins connected to MMPs’ expression, as well as elevated migration and invasion ability of CAFs in comparison to normal fibroblasts, and these features may stimulate cancer progression (Fig. 9), therefore a combination therapy against melanoma and melanoma-associated fibroblasts might be promising.

## Supplementary Information

**Additional file 1: Figure S1.** Comparison of CAFs derived upon TGFβ treatment or cultured in the presence of melanoma CM. Representative results of Western blot analysis using antibodies directed against αSMA, FAPα, pERK, and ERK are shown. Control for cells treated with TGFβ (CTRL TGFβ) constitutes of fibroblasts cultured in the presence of TGFβ solvent (acetonitrile). In the case of CAFs obtained using melanoma CM fibroblasts cultured in the mixture of FBM:DMEM (1:1) media were used as a control (CTRL CM).

**Additional file 2: Figure S2.** The proliferation rate of CAFs. Cell proliferation was measured using XTT assay. CAFs were obtained using melanoma conditioned media (CM) or in co-culture with melanoma cells on Transwell inserts (INS). Control (CTRL) constitutes of fibroblasts cultured in FBM:DMEM (1:1) medium. The data are shown as the mean ± SD.

**Additional file 3: Figure S3.** Measurement of CAFs Gelatin-FITC digestion activity. (A) General workflow for the analysis of Gelatin-FITC digestion activity. Left panel, upper row: Gelatin-FITC digested areas were detected (outlined in blue) and then measured (regions in black). Lower row: F-actin stain was used to mask a cell and determine its area (outline in red, filled in green). On the right: outlined in blue Gelatin-FITC digested areas overlapped with cell`s mask outlined in red.

## Data Availability

The datasets generated and/or analyzed during the current study are available from the corresponding author on reasonable request.

## Abbreviations

BCA: Bicinchoninic acid
bFGF: Basic fibroblasts growth factor
CAFs: Cancer-associated fibroblasts
CCL2: C-C motif chemokine ligand 2
CD44: Cluster of differentiation 44
CM: Conditioned medium
CTRL: Control
CXCL1: C-X-C motif chemokine ligand 1
DMEM: Dulbecco’s Modified Eagle Medium
DPPIV: Dipeptidyl peptidase IV
ECM: Extracellular matrix
EMT: Epithelial-mesenchymal transition
ERK: Extracellular signal-regulated kinase
FAP-α: Fibroblasts-activation protein alpha
FBM: Fibroblast Growth Basal Medium
FBS: Fetal bovine serum
FSP-1: Fibroblasts-specific protein 1
GM-CSF: Granulocyte–macrophage colony-stimulating factor
HGF: Hepatocyte growth factor
ICAM1: Intercellular adhesion molecule 1
IGFB-2: Insulin-like growth factor binding protein 2
IL6: Interleukin 6
INS: Transwell inserts
MAPK: Mitogen-activated protein kinase
MIF: Macrophage migration inhibitory factor
MMPs: Metalloproteinases
NHDF: Normal human dermal fibroblasts
PDGF A: Platelet-derived growth factor A
PIGF: Placental growth factor
PTX3: Pentraxin 3
RUNX2: Runt-related transcription factor 2
SDS: Sodium dodecyl sulfate
TIMP-4: Tissue inhibitor of metalloproteinases 4
TGFβ: Transforming growth factor-beta
TSP-1: Thrombospondin 1
uPA: Urokinase-type plasminogen activator
VEGFA: Vascular endothelial growth factor A
α-SMA: Alpha-smooth muscle actin
