# Supplementary figures and images for: Melanoma cells with diverse invasive potential differentially induce the activation of normal human fibroblasts
Source: Cell Commun Signal. 2022 May 10;20:63. doi: 10.1186/s12964-022-00871-x (PMC9092709; doi:10.1186/s12964-022-00871-x)

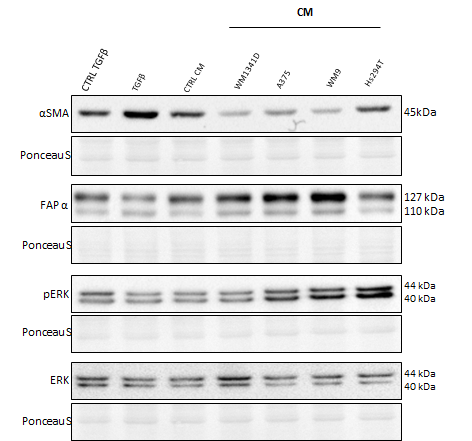

Supplement: Supplementary file 2 — Additional file 1: Figure S1. Comparison of CAFs derived upon TGFβ treatment or cultured in the presence of melanoma CM. Representative results of Western blot analysis using antibodies directed against αSMA, FAPα, pERK, and ERK are shown. Control for cells treated with TGFβ (CTRL TGFβ) constitutes of fibroblasts cultured in the presence of TGFβ solvent (acetonitrile). In the case of CAFs obtained using melanoma CM fibroblasts cultured in the mixture of FBM:DMEM (1:1) media were used as a control (CTRL CM). [file 12964_2022_871_MOESM2_ESM.tif]

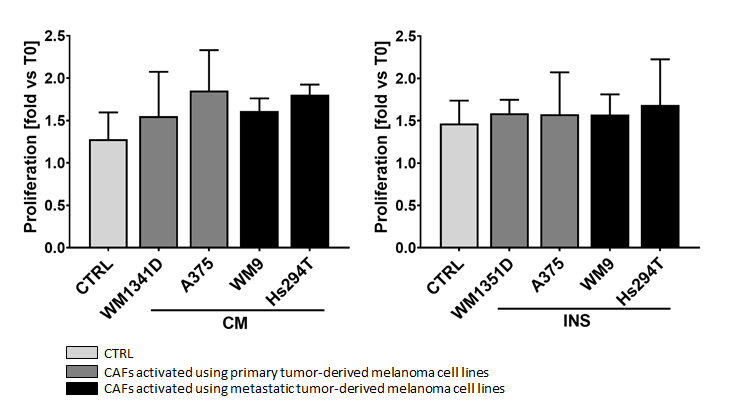

Supplement: Supplementary file 3 — Additional file 2: Figure S2. The proliferation rate of CAFs. Cell proliferation was measured using XTT assay. CAFs were obtained using melanoma conditioned media (CM) or in co-culture with melanoma cells on Transwell inserts (INS). Control (CTRL) constitutes of fibroblasts cultured in FBM:DMEM (1:1) medium. The data are shown as the mean ± SD. [file 12964_2022_871_MOESM3_ESM.tif]

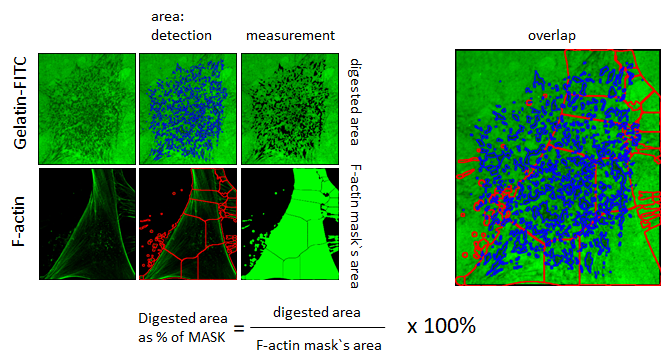

Supplement: Supplementary file 4 — Additional file 3: Figure S3. Measurement of CAFs Gelatin-FITC digestion activity. (A) General workflow for the analysis of Gelatin-FITC digestion activity. Left panel, upper row: Gelatin-FITC digested areas were detected (outlined in blue) and then measured (regions in black). Lower row: F-actin stain was used to mask a cell and determine its area (outline in red, filled in green). On the right: outlined in blue Gelatin-FITC digested areas overlapped with cell`s mask outlined in red. [file 12964_2022_871_MOESM4_ESM.tif]
